# Affordable web-based foot–ankle exercise program proves effective for diabetic foot care in a randomized controlled trial with economic evaluation

**DOI:** 10.1038/s41598-024-67176-6

**Published:** 2024-07-12

**Authors:** Ronaldo H. Cruvinel-Júnior, Jane S. S. P. Ferreira, Jady L. Veríssimo, Renan L. Monteiro, Érica Q. Silva, Eneida Y. Suda, Isabel C. N. Sacco

**Affiliations:** 1 Department of Physical Therapy, Speech, and Occupational Therapy, Faculdade de Medicina da Universidade de São Paulo, Rua Cipotânea, 51 - Cidade Universitária, São Paulo, São Paulo 05360-160 Brazil; 2 Department of Biological and Health Science, Universidade Federal do Amapá, Macapá, Amapá Brazil; 3 Masters and Doctoral Programs in Physical Therapy, Universidade Cidade de São Paulo, São Paulo, São Paulo Brazil

**Keywords:** Cost-effectiveness, Diabetic neuropathies, Foot exercise therapy, Rehabilitation technology, Prevention, Health care economics, Diabetes complications, Rehabilitation

## Abstract

The aim of this study was to shed light on a crucial issue through a comprehensive evaluation of the cost-effectiveness and cost-utility of a cutting-edge web-based foot–ankle therapeutic exercise program (SOPeD) designed for treating modifiable risk factors for ulcer prevention in individuals with diabetes-related peripheral neuropathy (DPN). In this randomized controlled trial, 62 participants diagnosed with DPN were assigned to the SOPeD software or received usual care for diabetic foot. Primary outcomes were DPN symptoms and severity, foot pain and function, and quality-adjusted life years (QALYs). Between-group comparisons provided 95% confidence intervals. The study also calculated incremental cost-effectiveness and cost-utility ratios (ICERs), analyzed direct costs from a healthcare perspective, and performed a sensitivity analysis to assess uncertainty. The web-based intervention effectively reduced foot pain, improved foot function and showed favorable cost-effectiveness, with ICERs ranging from (USD) $5.37–$148.71 per improvement in different outcomes. There is a high likelihood of cost-effectiveness for improving DPN symptoms and severity, foot pain, and function, even when the minimum willingness-to-pay threshold was set at $1000.00 USD. However, the intervention did not prove to be cost-effective in terms of QALYs. This study reveals SOPeD's effectiveness in reducing foot pain, improving foot function, and demonstrating cost-effectiveness in enhancing functional and clinical outcomes. SOPeD stands as a potential game-changer for modifiable risk factors for ulcers, with our findings indicating a feasible and balanced integration into public health systems. Further studies and considerations are vital for informed decisions to stakeholders and the successful implementation of this preventive program on a larger scale.

*Trial Registration*: ClinicalTrials.gov, NCT04011267. Registered on 8 July 2019.

## Introduction

The high prevalence of diabetes mellitus (DM) globally, set to increase in the future, is alarming^1^. Diabetes-related peripheral neuropathy (DPN), a prevalent and chronic DM complication, affects 12–50% of patients 10–15 years post-diagnosis^2^. DPN damages peripheral nerves, causing sensorimotor disorders and musculoskeletal deficits^3^, impacting muscle strength^4^, joint mobility^5^, consequently affecting the biomechanics of locomotor tasks^6,7^ and reducing the functionality and quality of life (QOL) for those afflicted^8^.

Beyond the human toll, DM-related expenditures worldwide are staggering, with a projected global cost of $745 billion by 2030^1^. In Brazil, the economic burden totaled $2.15 billion in 2016, including not only direct medical expenses, but indirect costs related to premature deaths, absenteeism, and early retirement^9^. The financial impact of foot complications arising from DPN, such as ulcers and amputations, strains healthcare systems and economies^10^, with an estimated annual cost of $128million in Brazil^11^. Thus, effective preventive strategies and innovative treatments for DM-related chronic complications are vital to reducing societal and economic burdens.

Recent International Working Group on the Diabetic Foot (IWGDF) guidelines recommended foot–ankle exercise programs as an effective strategy for preventing foot ulcers in people with DPN by modifying risk factors^12^. E-health programs, including telerehabilitation and web-based interventions, can be effective tools for foot–ankle exercise programs implementation as they addresses adherence issues like low motivation, high costs, and long durations of treatments^13,14^. Embracing the current trend of encouraging self-care and self-management among patients, our research group developed a cutting-edge, free, and public software application called the Diabetic Foot Guidance System (Sistema de Orientação ao Pé Diabético, or SOPeD; www.soped.com.br)^15^, offering a customized foot–ankle exercise program to prevent DM and DPN-related musculoskeletal and sensorial deficits, targeting ulcer risk factors. SOPeD's convenience, affordability, and accessibility make it an ideal option for widespread implementation.

To ensure that healthcare resources are allocated efficiently, especially in public health systems around the globe, and ensure that health outcomes are maximized, an economic evaluation of foot–ankle exercise programs compared with standard care is imperative. This would enable informed decisions by healthcare providers and policymakers, optimizing resource allocation for enhanced healthcare delivery^16,17^. Ensuring optimal care for individuals with DM and DPN, this evaluation is crucial for integrating innovative technologies like SOPeD into healthcare systems, benefiting a broader population and reducing burdens on individuals and healthcare systems.

Despite numerous low-risk bias studies advocating foot–ankle exercise programs as preventive strategies for ulcer risk factors^18^, economic evaluation is notably absent. Such evaluation is necessary to further validate and implement this therapeutic approach worldwide. Our aim in this study was to assess the cost-effectiveness and cost-utility of a 12-week SOPeD exercise program for changing modifiable risk-factors for ulcers in individuals with DM and DPN from a healthcare systems perspective.

## Materials and methods

### Study design

We conducted a superiority single-blind, randomized controlled trial (RCT) with two parallel arms (FOotCAre trial I) and an economic evaluation. This research project was approved by the Ethics Committee of the School of Medicine, University of São Paulo (CAAE#90,331,718.4.0000.0065). All procedures were carried out in compliance with the Declaration of Helsinki, and informed consent was obtained from all study participants. Furthermore, the RCT was prospectively registered at ClinicalTrials.gov on July 8, 2019 (NCT04011267), in accordance with the CONSORT guidelines. The economic evaluation reporting followed the Consolidated Health Economic Evaluation Reporting Standards^19^. The trial methodology and protocol have been extensively explained elsewhere^20^. Briefly, 62 eligible patients with DPN and categorized as IWGDF risk category 1 or 2^21^ were randomized 1:1 to receive either the SOPeD intervention (Intervention Group; IG) or usual care for 12 weeks (Control Group; CG) using a computer-generated permuted block randomization sequence^22^ in opaque, sealed envelopes by an independent researcher. Data confidentiality was ensured by compliance with the Brazilian General Data Protection Law (#13.709/2018). Allocation remained blinded throughout the study from the statistician and two researchers responsible for all assessments. Participants were assessed at baseline, after 12 weeks of intervention, and 24 weeks of followup, at the Physical Therapy, Speech and Occupational Therapy Department of the School of Medicine of the University of São Paulo.

### Participants’ recruitment and eligibility assessment

Participants were recruited between August/2019 and February/2022 through the database of the Endocrinology Outpatient Clinic of the Hospital das Clínicas (School of Medicine, University of São Paulo), and via a population campaign organized by the Brazilian Diabetes Care Association. The eligibility criteria were: adults of both sex, aged 18 to 65 years, with a clinical diagnosis of type 1 or 2 DM, who could walk independently, have access to internet, ability to use electronic devices, and a DPN severity score above 2, as confirmed by the Decision Support System for Classification of Diabetic Polyneuropathy^23^ (www.usp.br/labimph/fuzzy). The non-inclusion criteria were foot amputation; non-healed ulcer (> 6 months); history of foot–ankle, knee, or hip surgery; lower-limb arthroplasty and/or orthosis; non-DM-related neurological disease; dementia; receiving physiotherapy or offloading devices during the intervention; or major vascular complications and/or severe retinopathy. The principal investigator explained the study, risks, and benefits, and eligible participants signed informed consent approved by the Ethics committee.

### Treatment arms

The CG participants were provided with the usual care for diabetic foot, which consisted of the treatment suggested by the medical team, standard pharmacological intervention, self-care education, and self-management instructions based on the IWGDF^21^. A face-to-face consultation with a healthcare professional was carried out and a customized version of the self-care instructions, incorporating educational and self-management advice, was printed on a flyer and delivered to all participants^24^.

The IG participants were provided with the usual care, and also the SOPeD web-based foot–ankle exercise program^15^, three sessions per week for 12 weeks (total of 36 sessions). Each session lasted 20–30 min and included eight functional, stretching, and strengthening exercises for the extrinsic and intrinsic foot muscles. Exercise intensity and complexity was tailored to each individual's perceived effort, as entered by them into the software^15^. SOPeD also incorporates gamification components to enhance adherence and motivate users to maintain their exercise routine. Additionally, it encourages individuals to undergo regular reassessments every 30 days to ensure the execution and continuation of the exercise protocol. More information about the full trial protocol and the software’s components and structure can be found elsewhere^15,20^.

In the first face-to-face session, IG participants were guided on SOPeD use, exercise execution, and provided materials for performing the exercises. The main physical therapist remotely monitored the subsequent 35 sessions, contacting the participants by phone every week, tracking software access, and exercise frequency. Participants were instructed to report adverse events and discontinue in case of pain, excessive fatigue or discomfort. The participants were monitored throughout the 12-week program and were encouraged to continue using the SOPeD during the followup period.

### Outcome measures

The economic evaluation was conducted alongside the RCT that assessed multiple DPN-related and biomechanical outcomes. An analysis of effectiveness will be published, encompassing all these outcomes. The economic evaluation focused on reporting outcomes related to DPN, chosen as primary outcomes in the RCT due to their significant clinical relevance for individuals with DPN. Additionally, we incorporated the Quality-Adjusted Life Years (QALYs) measure, widely used for cost-utility analysis.

Effectiveness outcomes were measured and reported at 12 and 24 weeks, while cost-effectiveness and cost-utility were calculated at 24-week time-horizon, considering the entire study period. For the cost-effectiveness analysis, the primary outcomes were DPN symptoms assessed by the Brazilian version of the Michigan Neuropathy Screening Instrument (MNSI-BR), with scores ranging from 0 to 13 (with 13 representing the worst DPN)^25^; and DPN severity determined by the fuzzy score, ranging from 0 to 10, with a higher fuzzy score indicating more severe DPN.^23^. The secondary outcomes included foot-pain and foot-function scores from the Brazilian version of the Foot Health Status Questionnaire (FHSQ-BR); scores ranged from 0 to 100, where 100 represents the best condition and 0 the worst^26^.

For the cost-utility analysis, we calculated utility values based on the EuroQoL (EQ-5D-3L) questionnaire^27^, which includes five dimensions, each of which is rated by the participant on a three-level scale (no problems, some problems, and major problems). An algorithm, using population-based health preferences^28^, combined the responses for each dimension to yield a unique health-state score, ranging from − 0.59 to 1.00 (− 0.59 represents the worst possible health state, worse than death, while 1.00 represents perfect health), designated the utility value. Utility values were calculated for each assessment (baseline, 12 and 24-week). Preferences in cost-utility analysis refer to the relative values assigned to different health states by individuals or a specific population, reflecting their perceived desirability/utility of a particular health state. The increase in QALYs at 24-week followup was determined by computing the area under the curve of the utility values and assuming a linear change between each available time point.

### Resource use, valuation, and unit costs

The economic analysis was carried out from a healthcare systems perspective, in accordance with the Methodological Guidelines for Economic Evaluation by the Brazilian Ministry of Health^29^, for implementing any new health strategy in the Brazilian Public Healthcare Unified System (Sistema Único de Saúde; SUS).

Direct costs covered by SUS were calculated based on SUS reimbursement tables^30^. The index year for the analysis was 2022, and all expenses were converted from Brazilian reals to USD using purchasing power parities^31^. Discounting costs was not required due to the limited 24-week time-horizon duration^17^.

The healthcare costs encompassed all healthcare services, including medication. The intervention costs encompassed the cost of a face-to-face consultation with a healthcare professional for instructions about self-care and self-management (both groups), the first face-to-face session with a physiotherapist, and the cost of the exercise materials, as well as expenses associated with the hosting server, website, and app maintenance, which were estimated proportionally over a 24-week period. Details on costs and valuation per category of resources are presented in TableS1.

**Table 1:** Control and intervention groups clinical, demographic, and anthropometric outcomes at baseline (n = 62).

	Intervention group (n = 31) Mean ± SD	Control group (n = 31) Mean ± SD
Age (years)	52.1 ± 9.3	57.0 ± 9.6
Body mass (kg)	78.8 ± 13.4	85.7 ± 16.3
Height (cm)	167.0 ± 0.1	165.0 ± 0.1
Body mass index (kg/m^2^)	28.2 ± 4.1	31.7 ± 6.9
Female sex, n (%)	20 (64%)	18 (58%)
Type 2 diabetes, n (%)	26 (84%)	30 (97%)
Time of diabetes onset (years)	15.3 ± 9.4	10.3 ± 6.7
Tactile sensitivity (number of areas with loss of protective sensation)^a^	0 [0–1]	0 [0–0]
Vibration perception n (%)		
Absent	8 (26%)	6 (19%)
Reduced	4 (13%)	5 (16%)
Present	19 (61%)	20 (65%)
Recruitment location n (%)		
University tertiary hospital—Hospital das Clínicas	13 (42%)	15 (48%)
Local and regional population campaign	15 (48%)	11 (36%)
Brazilian Diabetes Care Association	3 (10%)	5 (16%)
Education n (%)		
Elementary education incomplete	2 (6%)	2 (6%)
Elementary education complete	1 (3%)	4 (13%)
High school incomplete	1 (3%)	3 (10%)
High school complete	12 (39%)	10 (33%)
Higher education incomplete	4 (13%)	5 (16%)
Higher education complete	11 (36%)	7 (22%)
Socioeconomic status n (%)		
Less than 1X Brazilian minimum salary/ month	1 (3%)	4 (13%)
1-3 x Brazilian minimum salary/ month	14 (45%)	19 (61%)
3-5 x Brazilian minimum salary/month	5 (16%)	5 (16%)
Over 5 x Brazilian minimum salary/month	11 (36%)	3 (10%)
DPN symptoms (MNSI score)	6.7 ± 1.9	6.3 ± 1.5
DPN severity (Fuzzy score)	3.9 ± 2.1	3.7 ± 2.0
EQ-5D (score)	0.6 ± 0.1	0.6 ± 0.1
FHSQ (score)		
Foot pain	46.7 ± 23.0	43.1 ± 25.5
Foot function	70.2 ± 26.5	63.0 ± 29.5

Data are presented as mean (SD) or as n or %

*SD* Standard Deviation; *MNSI* Michigan Neuropathy Screening Instrument; *FHSQ* Foot Health Status Questionnaire; *DPN* diabetes-related peripheral neuropathy; *EQ-5D* EuroQoL Questionnaire.

^a^Data is presented as median (interquartile range).

Costs were carefully assessed through a structured questionnaire, continuously from baseline to 24 weeks. Direct costs, including expenses related to medical consultation, medication, and any required ulceration treatments, were valued using Brazilian standard prices^30^. For medication, industry prices (lowest-priced option chosen) were used, with a sales tax rate of 18% applied in São Paulo.

### Statistical analysis

#### Sample size

The sample size was calculated using DPN symptoms as the primary outcome^20^. A statistical power of 0.80, alpha of 0.05, and effect size of 0.20 were used for the sample size calculation. The resulting sample size was 52 individuals. A final sample size of 62 patients was then chosen after estimating a dropout rate of 20%.

#### Effectiveness

The study employed an intention-to-treat approach and the Generalized Estimating Equation (GEE) method, using a gamma distribution and exchangeable correlation structure. Fixed factors included groups (CG and IG), assessment timepoints (baseline, 12 weeks, and 24 weeks), and the interaction effect (group-time). Normality of data distribution was confirmed by the Shapiro–Wilk test (*p* < 0.05). Missing data were assumed to be completely at random, as no discernible pattern in patient absences was observed during followup visits. We assessed our missing data through visual inspection, finding no systematic patterns. We chose GEE for its robustness in handling missing data and maintaining statistical efficiency even if the missing data are not completely at random. Between-group differences at 12 and 24 weeks, with corresponding 95% CIs, were reported. Statistical analyses utilized SPSS v.22.0 (IBM, Armonk, New York) with a significance level of 5%. The effectiveness analyses reported in this paper serve mainly to support the sensitivity analysis and provide readers with an overview of the conducted effectiveness analysis. The complete effectiveness analysis, encompassing all outcomes, will be published in a separate paper.

#### Cost-effectiveness and cost-utility analyses

Cost-effectiveness analysis was conducted using DPN symptoms and severity, foot pain and function, as outcome measures. Cost-utility analysis used QALYs as the outcome. Multiple imputation by chained equations was employed to address missing data in the cost-effectiveness analyses^32^. The imputation model included age, sex, body mass index, DM type, and all available baseline and follow-up cost and effect measure values. Ten complete datasets were created, resulting in a loss of efficiency of less than 5%. Pooled estimates were calculated according to Rubin’s rules^33,34^.

Over the 24-week time horizon, costs were accumulated and groups were compared using a non-parametric bootstrapping method, which provided 95%CI. The incremental cost-effectiveness and cost-utility ratios (ICERs) were calculated by dividing the difference in the total costs between treatments by the difference in the effects, following an intention-to-treat approach (base-case). To assess uncertainty, bootstrapping with 1000 replications was used and the results were presented in cost-effectiveness planes^35^.

A deterministic sensitivity analysis was performed to evaluate the robustness of the results, with cost-effectiveness and cost-utility analyses conducted under different scenarios. The first sensitivity analysis was based on a per-protocol approach, excluding patients with a treatment compliance rate < 70%. In the second sensitivity analysis, the outcomes obtained in the IG were changed up and down based on the lower and upper bounds of the 95%CI. Finally, in the third analysis, the costs in the IG were increased based on the upper bound of the 95%CI.

A probabilistic sensitivity analysis was also conducted, where cost-effectiveness acceptability curves were generated to determine the probability of each intervention being more cost-effective than the other at different levels of willingness-to-pay^36^. These cost-effectiveness acceptability curves considered the ICER values from the base-case and the ICER values from each scenario in deterministic sensitivity analyses. Calculations of the economic evaluation were conducted using the algorithms proposed by Gray et al.^37^, in Excel 2013.

### Data monitoring

A steering committee monitored the study's data, consisting of two graduate students (responsible for blind assessment, data tabulation, and coding), a senior researcher (overseeing data acquisition and data tabulation), a coordinator (managing the project), and an assistant researcher (handling participant recruitment and scheduling collections). Team members who were blinded gathered information using both physical and electronic forms to participants' allocation. The steering committee ensured the data's integrity and validity through edit checks at the time of data entry.

## Results

Participant flow, followup assessment visit attendance, and reasons for dropout are detailed in Figure S1. Both groups exhibited similar baseline characteristics and outcomes (Table 1). In the IG, 84% successfully completed the 12-week SOPeD program (26 participants). The dropout rate, defined as failure to attend both 12-week and 24-week assessments, was 19% in the IG (six participants) and 16% in the CG (five participants), totaling 18%. Some participants from both groups missed assessments due to the COVID-19. Moreover, one participant in the IG did not attend the 24-week assessment, and one participant in the CG did not attend the 12-week assessment. Additionally, two participants in the CG did not attend the 24-week assessment. Two IG patients reported mild adverse effects during the intervention (delayed-onset muscle soreness and foot muscle cramping), with no withdrawals from the RCT due to these effects.

Mean healthcare costs were similar for both groups (difference, USD$67.11, 95%CI -USD$52.69, USD$189.82; Table 2). The cost of treating ulceration in the IG was zero, because no IG participant developed ulceration during the study period.

**Table 2:** Mean costs (In USD).

Cost type	Mean per group		Mean difference (95% CI)^a^	*p*-value
	Intervention Group (n = 31)	Control Group (n = 31)		
Medical consultation (specialized medical care)	4.85	6.90	− 2.02 (− 4.60, 0.51)	0.109
Medication costs	310.11	290.46	18.94 (− 105.11, 150.42)	0.771
Treatment of ulceration	0.00	4.11	− 4.08 (− 12.32, 0.00)	0.321
Intervention Costs (SOPeD versus Usual Care)	54.78	2.49	52.29 (52.29, 52.29)	0.001*
Total	371.13	303.97	67.11 (− 52.69, 189.82)	0.322

^a^The upper and lower confidence limits at the 2.5th and 97.5th percentile based on 1000 bootstrap replications.

*Indicates a significant difference (p < 0.05) between groups.

The reported costs encompass the costs over 24 months.

The effectiveness of the web-based intervention resulted in a noteworthy reduction in foot pain and an improvement in foot function in the IG compared to the CG. As depicted in Table S2, at the 24-week, foot-pain scores showed an improvement of 20.2 points (95%CI 4.0–36.3), and foot-function scores significantly enhanced 18.5 points (95%CI 3.9–33.0). These findings highlight the effectiveness of the SOPeD in positively modifying these clinical outcomes.

**Figure 1:**
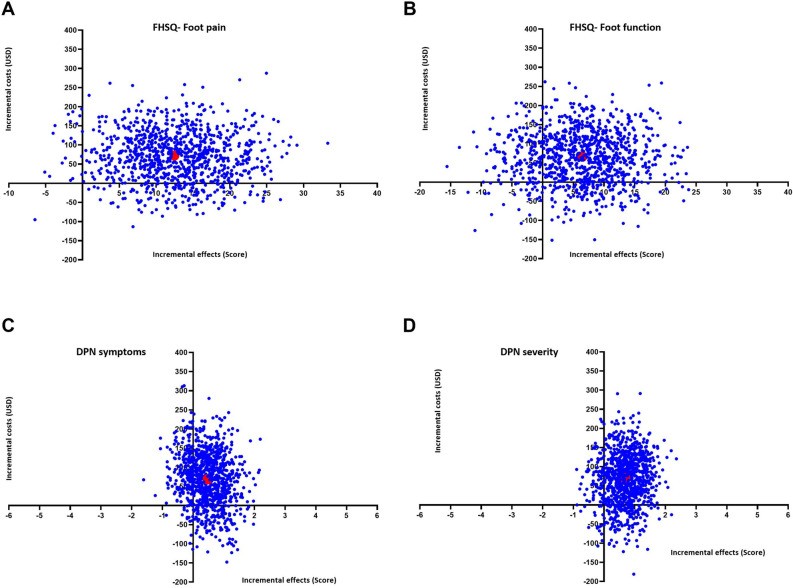
Cost-effectiveness planes for different outcomes: (**A**) foot pain, (**B**) foot function, (**C**) DPN symptoms, (**D**) DPN severity. The x-axis represents the incremental level of effectiveness of the outcome (incremental effects), while the y-axis represents the additional total costs (incremental costs). The red dots represent the mean ICER and the blue dots represent the ICERs from a bootstrapping technique with 1000 replications. DPN, diabetes-related peripheral neuropathy; ICERs, incremental cost-effectiveness and cost-utility ratios; USD, United States dollars; FHSQ, foot health status questionnaire.

With regard to the cost-effectiveness, the ICER was USD$148.71 per point of DPN symptoms score, USD$102.26 per point of DPN severity score, USD$5.37 per point of foot pain score, and USD$10.54 per point of foot function score (Table 3, Fig. 1), indicating that the costs for improvement in DPN symptoms and severity, foot pain, and foot function are relatively low. The intervention (SOPeD) exhibited slightly higher incremental costs, along with the greater incremental effects, compared with usual care.

**Table 3:** Cost-effectiveness and cost-utility analyses (ICERs) at 24 weeks after the start of the intervention.

Outcome	Incremental costs (95%CI) (USD)	Incremental effects (95%CI) (Score)	ICER (USD/Score)
DPN symptoms (0 to 13)	67.16 (− 6.22, 140.54)	0.45 (0.16, 1.06)	148.71
DPN severity (0 to 10)	67.16 (− 6.22, 140.54)	0.66 (0.10, 1.21)	102.26
QALY (0 to 1)	67.16 (− 6.22, 140.54)	− 0.07 (− 0.11, − 0.02)	− 1018.19
Foot pain (0 to 100)	67.16 (− 6.22, 140.54)	12.50 (5.66, 19.34)	5.37
Foot function (0 to 100)	67.16 (− 6.22, 140.54)	6.37 (0.91, 13.66)	10.54
Sensitivity analysis per protocol (deterministic analysis)			
DPN symptoms (0 to 13)	67.16 (− 6.22, 140.54)	0.40 (− 0.28, 1.09)	266.64
DPN severity (0 to 10)	67.16 (− 6.22, 140.54)	0.36 (− 0.09, 0.80)	301.60
QALY (0 to 1)	67.16 (− 6.22, 140.54)	− 0.02 (− 0.07, 0.03)	− 5130.82
Foot pain (0 to 100)	67.16 (− 6.22, 140.54)	13.91 (5.81, 22.02)	7.75
Foot function (0 to 100)	67.16 (− 6.22, 140.54)	14.48 (5.93, 23.02)	7.32
Sensitivity analysis in which the effects were decreased based on the lower bound of the 95% CI (deterministic analysis)			
DPN symptoms (0 to 13)	67.16 (− 6.22, 140.54)	− 0.85 (− 1.23, − 0.48)	− 78.77
DPN severity (0 to 10)	67.16 (− 6.22, 140.54)	− 0.13 (− 0.46, 0.21)	− 529.76
QALY (0 to 1)	67.16 (− 6.22, 140.54)	− 0.14 (− 0.17, − 0.11)	− 492.62
Foot pain (0 to 100)	67.16 (− 6.22, 140.54)	4.38 (− 0.75, 9.51)	15.35
Foot function (0 to 100)	67.16 (− 6.22, 140.54)	− 1.11 (− 7.09, 4.86)	− 60.33
Sensitivity analysis in which the effects were increased based on the upper bound of the 95% CI (deterministic analysis)			
DPN symptoms (0 to 13)	67.16 (− 6.22, 140.54)	0.72 (0.34, 1.09)	93.61
DPN severity (0 to 10)	67.16 (− 6.22, 140.54)	1.44 (1.11, 1.78)	46.53
QALY (0 to 1)	67.16 (− 6.22, 140.54)	0.01 (− 0.03, 0.03)	18.315,02
Foot pain (0 to 100)	67.16 (− 6.22, 140.54)	20.62 (15.49, 25.75)	3.26
Foot function (0 to 100)	67.16 (− 6.22, 140.54)	13.85 (3.05, 19.82)	4.85
Sensitivity analysis in which costs were increased based on the upper bound of the 95% CI (deterministic analysis)			
DPN symptoms (0 to 13)	71.88 (15.77, 127.99)	0.45 (0.16, 1.06)	159.17
DPN severity (0 to 10)	71.88 (15.77, 127.99)	0.66 (0.10, 1.21)	109.45
QALY (0 to 1)	71.88 (15.77, 127.99)	− 0.07 (− 0.11, − 0.02)	− 1.089,77
Foot pain (0 to 100)	71.88 (15.77, 127.99)	12.50 (5.66, 19.34)	5.75
Foot function (0 to 100)	71.88 (15.77, 127.99)	6.37 (0.91, 13.66)	11.28

Data are presented as mean (95% CI).

*ICER* Incremental Cost Effectiveness Ratio; *CI* Confidence Interval; *USD* United States Dollars; *DPN* diabetes-related peripheral neuropathy; *QALY* Quality Adjusted Life Years.

**Figure 2:**
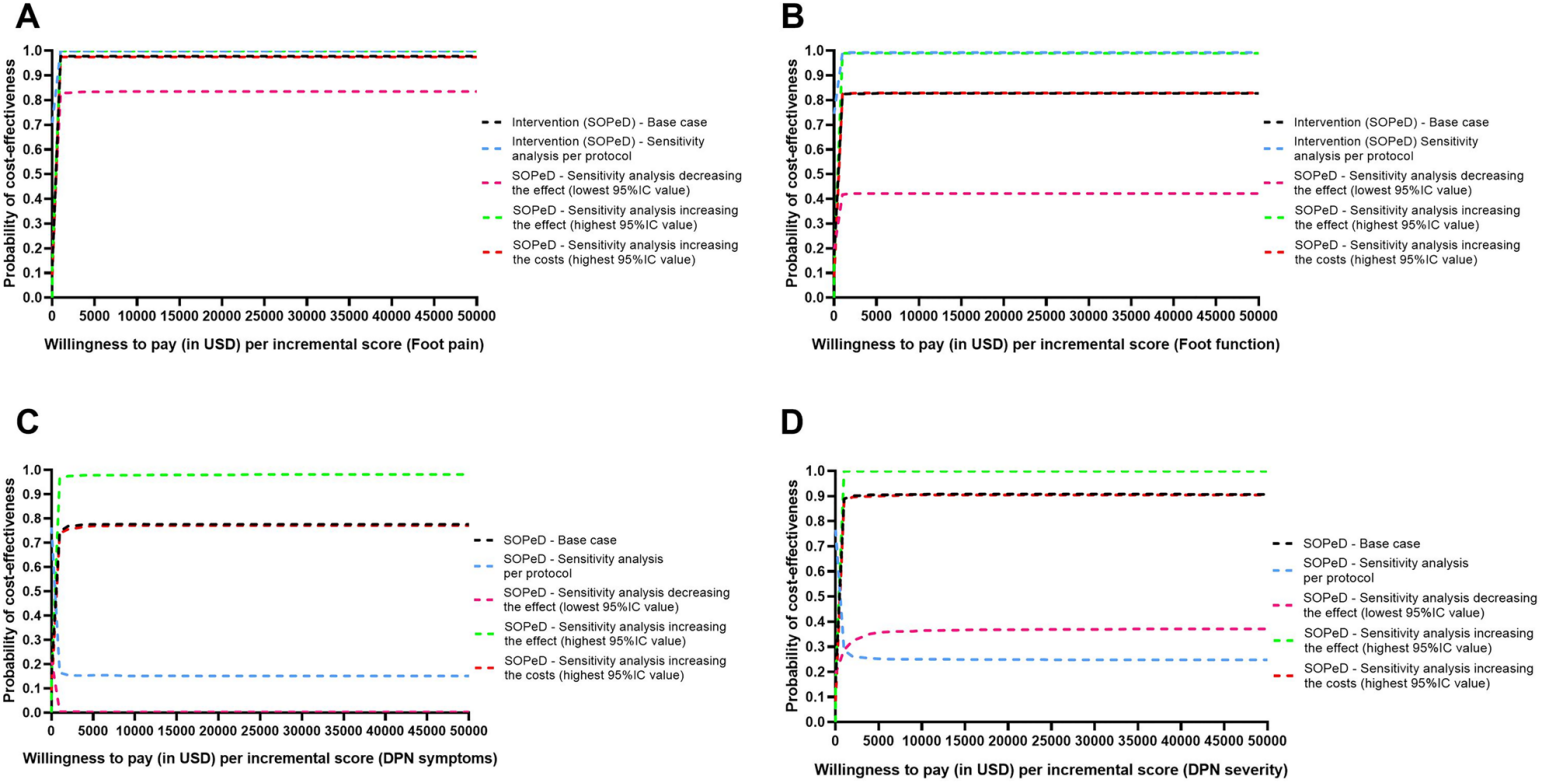
Acceptability curves (base-case and sensitivity analysis) of cost-effectiveness for different outcomes: (**A**) foot pain, (**B**) foot function, (**C**) DPN symptoms, (**D**) DPN severity. The x-axis represents the incremental level of effectiveness of the outcome (incremental effects), while the y-axis represents the additional total costs (incremental costs). USD, United States dollars; DPN, diabetes-related peripheral neuropathy; SOPeD, Sistema de Orientação ao Pé Diabético (web-based foot–ankle therapeutic exercise program).

The SOPeD intervention demonstrated a cost-effectiveness probability of 77.6% for DPN symptoms, 90.7% for DPN severity, 98.7% for foot pain, and 82.5% for foot function, even when considering a minimum willingness-to-pay threshold of USD$1000.00 (Fig. 2). However, the intervention did not show cost-utility for QALY (cost-utility probability of 44.0%). When varying the incremental effects through the per-protocol sensitivity analysis and increasing the costs based on the upper bound of the 95%CI, both scenarios revealed similar results to the base-case. The results of the sensitivity analysis performed by decreasing the effects of the intervention differed from the base-case (most of the outcomes became non-cost-effective), with only the foot-pain outcome remaining unchanged. The sensitivity analysis indicated that the positive effects of the intervention were similar to those in the base-case, except for the cost-utility for QALY, which became cost-utile, with a positive ICER (Table 3, Fig. 2).

## Discussion

As a part of the RCT studying the difference in effectiveness of SOPeD vs. usual care, an economic evaluation was conducted to assess the difference between them in cost-effectiveness and cost-utility. SOPeD was found to be modestly effective to change the main modifiable risk-factors for ulcers, easily implementable, and budget-friendly—undeniably cost-effective for DPN-related outcomes and foot-related aspects. The ICERs for reducing DPN symptoms and severity, reducing foot pain, and improving foot function were found to be remarkably low, demonstrating that these gains come at an affordable cost. Even at a minimum willingness-to-pay threshold of USD$1000.00, the intervention showed great potential for cost-effectiveness, especially for foot pain, and for DPN symptoms, severity, and foot function. Regrettably, SOPeD did not achieve cost-utility for QALY.

SOPeD was most cost-effective for foot pain and function. However, the cost-effectiveness analysis differed from the cost-utility analysis. QALY effects were similar in both interventions, with SOPeD costs slightly higher (hosting server, website, app maintenance). Although not statistically different from usual care, the incremental cost for gaining a QALY was higher for the SOPeD than for usual care, even in a per-protocol analysis. This prompts questions about the treatment's clinical significance or a lack of sensitivity to detect QOL improvements. The differences between generic health-related QOL measures and disease-specific measures have been discussed in previous studies^38^.

Sensitivity analyses consistently showed SOPeD's high cost-effectiveness across various scenarios, indicating a high probability of SOPeD being more cost-effective than usual care. Even under worst-case scenarios, increasing the costs and decreasing the incremental effects, SOPeD remained highly cost-effective for overall outcomes and for foot pain. In a more favorable scenario, increasing the incremental effects to mirror the results of previous RCTs with greater effects^39,40^, SOPeD proved to be cost-effective for all outcomes, including QALY.

SOPeD significantly improved foot pain and function compared to usual care, suggesting its effectiveness. A recent IWGDF meta-analysis found that foot–ankle exercise programs can improve ankle and first metatarsophalangeal joint mobility, as well as DPN signs and symptoms^18^. Nevertheless, the analyzed interventions lacked the incorporation of rehabilitation technology, such as web-based programs. Engaging in SOPeD web-based foot–ankle exercises appears to be as effective as face-to-face (individual or group-based) or home-based therapeutic programs.

Our point estimates in the cost-effectiveness analysis may slightly differ from those derived from the effectiveness analysis. This divergence can be attributed to the utilization of distinct statistical methods for these two analyses, as indicated by Raftery et al.’s findings^41^. Our study did not track participants' blood glucose and glycated hemoglobin levels, which could have affected the results of the primary outcomes related to DPN, such as burning pain, muscle cramps, and prickling sensations. Elevated blood glucose levels may intensify DPN symptoms, which would have also influenced the assessment of DPN severity, as the fuzzy classification took into account MNSI-BR scores.

Importantly, cost-effectiveness was analyzed from a healthcare systems perspective, as this technology is intended for use in the SUS. However, from a societal perspective, higher costs for the CG could lead to a further positive reduction in the incremental cost-effectiveness ratio for implementing SOPeD. The reasons for this are the following: (1) the IG resulted in no costs associated with ulcer treatment, as none of the participants developed ulcers during the study, whereas one CG participant did; and (2) the costs from a healthcare systems perspective exclude expenses related to work absenteeism and indirect costs, such as transportation to medical appointments, which are worthy of consideration from a societal perspective.

According to the recommendations of the National Commission for the Incorporation of Technologies in SUS^42^, the willingness-to-pay threshold in Brazil was set at R$40,000.00 reais (approximately USD$8000.00) per QALY gained. This threshold makes the implementation of SOPeD in SUS feasible, as it has demonstrated cost-effectiveness for the outcomes of foot pain and function, even when considering a minimum willingness-to-pay threshold of USD$1000.00. A threshold of USD$8000 (converted from Brazilian reais to USD) renders it highly viable. In other words, SOPeD represents an intervention with substantial effectiveness and minimal implementation costs, compared with usual care. Considering the similar costs in both interventions and Brazil’s R$40,000 allocations for technology incorporation, SOPeD can be readily integrated into the healthcare system, representing a cost-neutral addition with significant potential benefits. It is important to mention that the results of our economic evaluation, conducted in Brazil, may vary in different countries due to differences in medical consultation costs and healthcare system structures.

In addition to costs, there are other important aspects that must be taken into account when considering implementing SOPeD as a complementary treatment for people with DPN, both in Brazil and in other health systems worldwide. Firstly, there is currently no standard intervention for modifying musculoskeletal and somatosensory risk factors for foot ulcers in people with DPN and foot pain; SOPeD represents a feasible and potentially cost-effective strategy to address this need. Secondly, patient preferences play a pivotal role in healthcare decision-making. Our study demonstrated that the SOPeD technology exhibits high levels of acceptability and satisfaction among users, and all patients who tested this web-based intervention would readily recommend it to others with DM and DPN^13^. Thirdly, patients highly prioritize improvements in DPN-related symptoms and functionality, precisely the outcomes that proved cost-effective. Lastly, the observed balance between desirable and undesirable effects showed minimal adverse events and significant beneficial effects, as evidenced by improvements in foot function and reductions in foot pain and DPN severity and symptoms, further confirming the feasibility of SOPeD, as previously demonstrated^13^.

This trial has limitations. First, we relied on self-reported costs from patients, potentially introducing inaccuracies. However, due to the impracticality of alternatives, this method was necessary. Nevertheless, since the measurements were similar in both groups, the comparability should not be affected. A previous study has also demonstrated the acceptability of this measurement method^43^. Second, costs and effects were analyzed over a 24-week period. It is possible that the costs and effects may change beyond this timeframe, which could lead to different conclusions regarding cost-effectiveness in longer trials. Future research should consider conducting studies over longer durations after the intervention has ended.

In summary, the findings support SOPeD's effectiveness and economic viability in managing modifiable risk-factors for ulcers, positioning it as a promising and potentially impactful addition to healthcare interventions targeting foot-related complications associated with DPN. Moreover, this study provides valuable input for further modeling studies on long-term costs and effectiveness of foot–ankle exercise preventive programs.

## Conclusions

This study demonstrated that SOPeD resulted in significant improvements in foot pain, foot function, and DPN symptoms and severity, although there were no improvements in QALY. The program exhibited comparable costs to usual care and demonstrated cost-effectiveness for foot pain, foot function, and DPN severity and symptoms. SOPeD may be a promising preventive option for individuals with DPN to prevent modifiable risk-factors for ulcers, and its implementation in public health systems appears feasible, acceptable, and valuable, with a good balance between desirable and undesirable effects. However, further research is necessary to make informed decisions and effectively implement the program on a large scale.

### Supplementary Information

Supplementary Information 1.

Supplementary Information 2.

## Data Availability

The datasets used and/or analyzed in this study are available as anonymized data in the University's public repository (https://repositorio.usp.br/).
