# Supplementary figures and images for: Affordable web-based foot–ankle exercise program proves effective for diabetic foot care in a randomized controlled trial with economic evaluation
Source: Sci Rep. 2024 Jul 12;14:16094. doi: 10.1038/s41598-024-67176-6 (PMC11245594; doi:10.1038/s41598-024-67176-6)

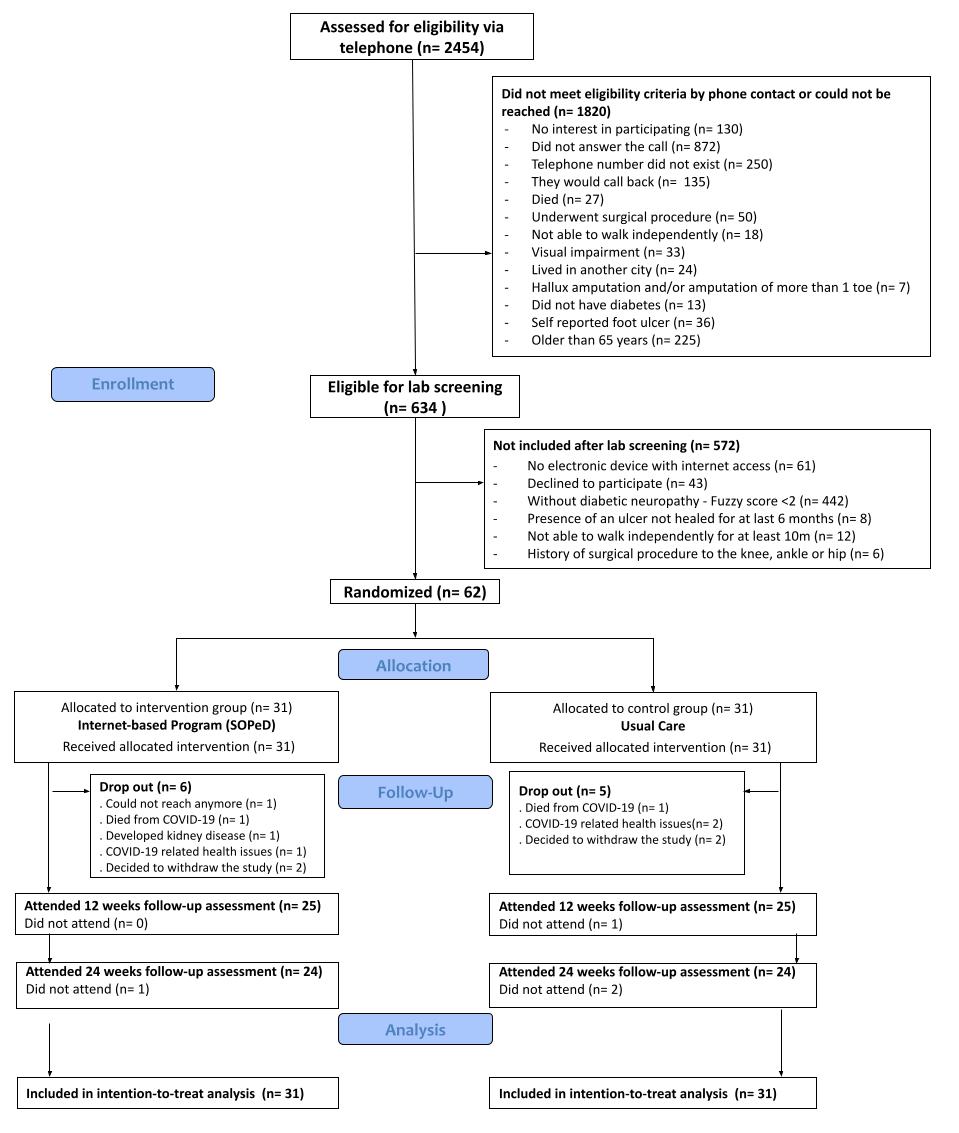

Supplement: Supplementary file 1 — Supplementary Information 1. [file 41598_2024_67176_MOESM1_ESM.jpg]
