# Supplementary material for: Affordable web-based foot–ankle exercise program proves effective for diabetic foot care in a randomized controlled trial with economic evaluation
Source: Sci Rep. 2024 Jul 12;14:16094. doi: 10.1038/s41598-024-67176-6 (PMC11245594; doi:10.1038/s41598-024-67176-6)

**Cost-effective approach for diabetic foot care: unleashing the potential of a web-based foot-ankle exercise program through a randomized controlled trial with economic evaluation**

Ronaldo H. Cruvinel-Júnior^1^, Jane S.S.P. Ferreira^1^, Jady L. Veríssimo^1^, Renan L. Monteiro^1,2^, Érica Q. Silva ^1^, Eneida Y. Suda^1,3^, Isabel C. N. Sacco^1*^

1. Department of Physical Therapy, Speech, and Occupational Therapy, Faculdade de Medicina da Universidade de São Paulo, São Paulo, Brazil.

2. Department of Biological and Health Science, Universidade Federal do Amapá, Amapá, Brazil.

3. Masters and Doctoral Programs in Physical Therapy, Universidade Cidade de São Paulo, São Paulo, Brazil, São Paulo, SP, Brazil.

**Supplementary Appendix**

**Content**

Supplementary tables ………………………………………………………………………………........................... 2

Supplementary Table S1 - Costs and valuation per category of resources ……………………… 2

Supplementary Table S2 - Estimated effect of SOPeD vs Usual Care (95%CI) at 12 and 24 weeks ……………………………………………………………………………………………………………………………………………..2

Supplementary figures ………………………………………………………………………………………..…….……….. 3

Supplementary Figure S1 - Flowchart of recruitment, assessment, and follow-up process of the randomized controlled trial …….…………………………………………………………………..…….……………….. 3

**Supplementary tables**

Supplementary Table S1 - Costs and valuation per category of resources

| **Type of resources** | **Unit** | **Valuation (in USD)** |
| --- | --- | --- |
| **Healthcare costs** |  |  |
| Medical consultation (specialized medical care) | Per consult | 3.96 |
| Medication costs | Per unit of medicine | * |
| Treatment of ulceration | Per treatment | 127.34 |
| **Intervention Costs** |  |  |
| Hosting Server Maintenance | Per 24/weeks | 62.25 |
| App and website maintenance | Per 24/weeks | 522.54 |
| Exercise kit containing materials needed to perform  the exercises | Per kit | 30.93 |
| Initial face-to-face session with a physical therapist | Per consult | 2.49 |
| Consultation with a healthcare professional for  instructions about self-care education and self-management | Per consult | 2.49 |

^*^ Costs for medication were calculated by taking the lowest price per unit of medicine in 2022. The reference medication prices were extracted from the 2022 Public Maximum Price List for Active Ingredients (CMED, 2022).

Supplementary Table S2 - Estimated effect of SOPeD vs Usual Care (95%CI) at 12 and 24 weeks

| **Outcome** | **Intervention Group**  **Mean (SD)**  **(n=31)** | **Control Group**  **Mean (SD)**  **(n=31)** | **Estimated effect of Intervention Group vs Control Group (95% CI)^a^** |
| --- | --- | --- | --- |
| **DPN symptoms (MNSI score)** |  |  |  |
| 12 weeks | 5.36 (2.75) | 5.35 (2.35) | 0.0 (-1.33 , 1.39) |
| 24 weeks | 5.00 (2.72) | 4.97 (1.83) | 0.4 (-1.6 , 0.8) |
| **DPN severity (Fuzzy score)** |  |  |  |
| 12 weeks | 3.23 (2.50) | 3.32 (1.83) | -0.1 (-1.3 , 1.2) |
| 24 weeks | 2.68 (2.23) | 3.07 (1.49) | -0.5 (-1.9 , 0.8) |
| **QALY (EQ-5D score)** |  |  |  |
| 12 weeks | 0.65 (0.16) | 0.62 (0.17) | 0.0 (-0.1 , 0.1) |
| 24 weeks | 0.64 (0.16) | 0.58 (0.17) | 0.0 (-0.0 , 0.1) |
| **Foot pain (FHSQ score)** |  |  |  |
| 12 weeks | 68.10 (25.53) | 50.16 (29.14) | 13.1 (-1.8 , 28.1) |
| 24 weeks | 65.85 (25.46) | 49.78 (30.57) | 20.2 (4.0 , 36.3)* |
| **Foot function (FHSQ score)** |  |  |  |
| 12 weeks | 76.14 (28.19) | 74.05 (26.76) | 2.08 (-13.63 , 17.79) |
| 24 weeks | 82.06 (23.48) | 68.55 (29.25) | 18.5 (3.9 , 33.0)* |

^a^ Estimated effect of Intervention Group versus Control Group: Values were calculated with Generalized Estimating Equation. * Indicates a statistically significant effect of p<0.05.

Supplementary Figure S1 - Flowchart of recruitment, assessment, and follow-up process of the randomized controlled trial


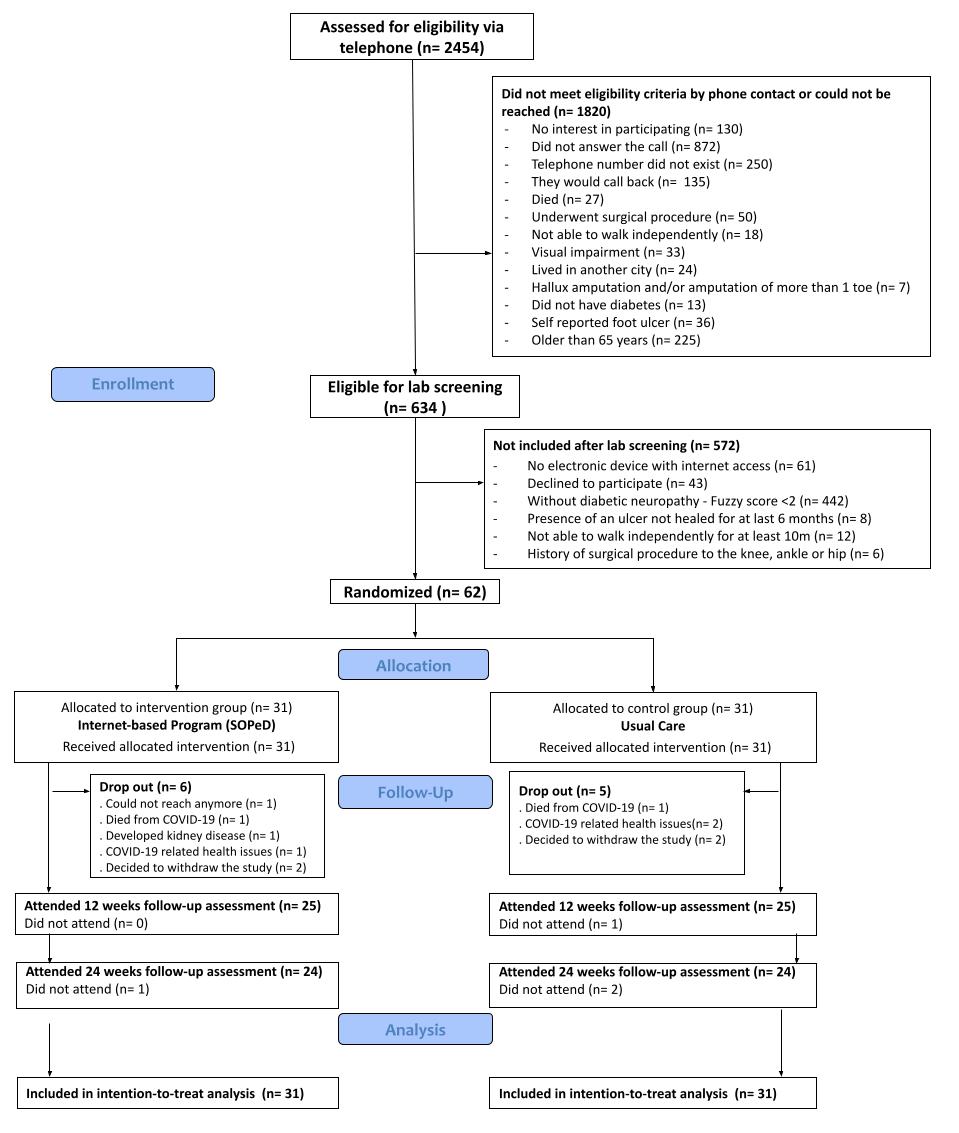

Supplement: Supplementary file 2 — Supplementary Information 2. [file 41598_2024_67176_MOESM2_ESM.docx]
